# Fatigue in cancer patients: comparison with the general population and prognostic factors

**DOI:** 10.1007/s00520-019-05260-8

**Published:** 2020-01-17

**Authors:** Andreas Hinz, Joachim Weis, Elmar Brähler, Martin Härter, Kristina Geue, Jochen Ernst

**Affiliations:** 1 Department of Medical Psychology and Medical Sociology, University of Leipzig, Philipp-Rosenthal-Str. 55, 04103 Leipzig, Germany; 2 Department of Psychooncology, Clinic of Cancer Rehabilitation UKF Reha gGmbH, University Clinic Centre Freiburg, Freiburg, Germany; 3 Department of Psychosomatic Medicine and Psychotherapy, University Medical Center Mainz, Mainz, Germany; 4 Department and Outpatient Clinic of Medical Psychology, University Medical Center Hamburg-Eppendorf, Hamburg, Germany

**Keywords:** Fatigue, Psychometrics, Cancer burden, Prognostic factors

## Abstract

**Purpose:**

The aims of this examination were to compare cancer patients’ fatigue burden with that of the general population, to identify clinical factors that are associated with fatigue, and to test psychometric properties of the fatigue questionnaire MFI-20 including the short-form MFI-10.

**Methods:**

A sample of 1818 German cancer patients was tested with the MFI-20.

**Results:**

The study confirmed that the cancer patients demonstrate a high level of burden from fatigue. The effect size for the comparison between the cancer patients and a sample of the general population (*n* = 1993) was *d* = 0.58 based on MFI-20 total scores. In the cancer patients’ sample, females reported slightly higher levels of fatigue than males did (*p* < 0.05). There was no significant effect of age on fatigue. Advanced tumor stage, the presence of metastases, and a “poorer” Eastern Cooperative Oncology Group (ECOG) performance status were significantly associated with fatigue. The results of the confirmatory factor analyses (CFAs) only partly confirmed the factorial structure of the MFI-20.

**Conclusion:**

Despite the insufficient CFA indices, we believe that the scale structure of the MFI-20 should not be changed and that calculating a total fatigue score is justifiable. For those seeking a shorter questionnaire, the MFI-10, which only contains those 10 items which positively indicate fatigue, is a good alternative.

## Introduction

Fatigue is one of the most common symptoms cancer patients experience [1, 2]. Cancer survivors are also often affected by this burdensome symptom [3]. In contrast to normal tiredness, fatigue cannot be relieved by common strategies known to restore energy [4]. In addition to cancer, heightened levels of fatigue can also be found in patients with other diagnoses such as cardiovascular diseases [5], lung diseases [6], and rheumatoid arthritis [7]. Multiple questionnaires have been developed to assess fatigue. A review paper summarizes 40 such instruments [8]. One often used instrument is the Multidimensional Fatigue Inventory MFI-20. This questionnaire was developed in 1995 in the Netherlands [9] and has been translated into English and many other languages, e.g., German [10], French [11], Swedish [12, 13], Spanish [14], Korean [15], Hindi [16], and Chinese [17]. General population studies have been performed with the MFI-20 in several countries [13, 14, 18]. There is a long-standing debate about the factorial structure of the MFI-20. Several examinations failed to confirm the five-dimensional structure (general fatigue, physical fatigue, reduced activity, reduced motivation, mental fatigue) that was proposed by the original test authors [11, 19–21]. A French study [19] retained all 20 items and combined two of the dimensions into one thereby resulting in four dimensions. Another study [11] restricted the analysis to 15 items and arrived at four dimensions. A Polish study also removed five items and assigned the remaining items to only three factors. The Swedish general population study [13] did not change the factorial structure, but the researchers only reported the results of four out of the five scales, the subscale reduced motivation was ignored due to bad psychometric properties. A study done among 1494 German cancer patients restricted the analyses to one out of the five dimensions, namely, general fatigue [22]. One putative reason for the poor fit indices is the fact that all MFI-20 scales include both two positively oriented and two negatively oriented items. Using items of different orientation can reduce the reliability of the scales [23, 24]. Therefore, Baussard et al. [25] developed a shortened version of the questionnaire, the MFI-10. It consists only of those 10 items which positively indicate fatigue and excludes the items with the opposite direction.

The main objectives of this paper were to test the MFI-20 in a large sample of German cancer patients and to examine factors associated with fatigue. In particular, the aims were (a) to examine cancer patients’ levels of fatigue—compared with those of the general population, (b) to analyze the impact of age, gender, and clinical factors (tumor site, time since diagnosis, ECOG performance status, presence of metastases, and setting) on fatigue, (c) to examine the relationship between the fatigue dimensions and other scales of quality of life (QoL), and (d) to test psychometric properties of the MFI-20 and the short-form MFI-10.

## Methods

### Cancer patients

This multicenter study included cancer patients receiving treatment in acute care hospitals, outpatient facilities, and rehabilitation clinics. The aim was to obtain as representative as possible a sample of German cancer patients. Five study centers in Germany contributed to this total project; three of them also included the MFI-20. The following institutions were involved at each study center: the local university hospital, at least one other maximum care hospital, at least two ambulatory facilities, and at least two rehabilitation clinics. Further details of the study methods have been described elsewhere [26]. Results of this study concerning the prevalence of mental disorders have already been published [27, 28]. Inclusion criteria were the presence of a malignant tumor and age between 18 and 75 years. Study candidates were excluded if they had a severe physical, cognitive, and/or verbal impairment that would interfere with their ability to give informed consent. Trained research assistants contacted the patients who fulfilled the inclusion criteria and asked them to participate. All participating patients provided written informed consent. The response rate was 68.1%. While the whole study comprised 4020 patients, in three of the five participating study centers (Leipzig, Hamburg, and Freiburg), some further questionnaires were included in addition to the core questionnaires which were administered to all patients. One of these additional questionnaires was the MFI-20. Therefore, MFI-20 data sets are available for a subsample of the 4020 patients. The study was conducted in accordance with the Declaration of Helsinki and was approved by the ethics committees of all participating centers.

### General population

The general population sample was derived from a general population survey [18]. Starting from more than 200 sampling points representing all regions of Germany, street, house, and flat were chosen via the random-route technique. Finally, the target person in the household was also selected randomly using the Kish-selection-grid technique. The response rate of this examination was 68%. The sample was fairly representative of the general population of Germany in terms of age, gender, and education. In total, the sample of the general population comprised *n* = 1993 people in the age range 18–93 years, 874 of whom were males and 1119 females.

Since the average age of the cancer patients’ sample was higher than that of the general population sample, we selected a subsample of the 1993 persons so that the age and gender distributions of that group were nearly identical to those of the patients. This was achieved by successively removing younger participants and females until the man age and gender distribution of the cancer patients was reached. The selected subsample comprised 1397 persons (630 males and 767 females; proportion of females, 54.9%) with a mean age of 58.5 years, which is nearly identical to the data points of the patients’ sample.

### Instruments

#### MFI-20

The MFI-20 consists of 20 items which belong to 5 dimensions. Each item has to be answered on a five-point Likert scale (range 1–5); the scale scores range from 4 to 20. An item example is: “I feel very active.” Each scale consists of two positively oriented items and two negatively oriented items. Although the authors of the original test did not recommend calculating a total score over all 20 items, it is possible to use such a sum score [29]. The shortened MFI-10, according to Baussard et al. [25], consists of the 10 positively oriented items (items 2, 5, 9, 10, 13, 14, 16, 17, 18, and 19) which indicate the presence of fatigue and omits those negatively oriented items which indicate the absence of fatigue. Though this MFI-10 can also be decomposed into three subscales [25], we use the MFI-10 as a 10-item scale in the descriptive analyses.

#### EORTC QLQ-C30

The QoL questionnaire EORTC QLQ-C30 [30] consists of 15 scales: five functioning scales (physical, role, cognitive, emotional, and social functioning), eight symptom scales, one item concerning financial difficulties, and one 2-item global QoL scale. One of the symptom scales is the three-item fatigue scale, an example item is: “Were you tired?” High functioning scores and low symptom scores indicate good QoL. It is also possible to use a summarizing score that averages across all functioning scores and all (inverted) symptom scores according to Giesinger et al. [31]. Normative values of the EORTC QLQ-C30 are available [32, 33].

### Statistical analyses

Effect sizes *d* were used to express the mean score differences between patients and the general population in relation to the standard deviations. A two-way ANOVA was performed to test the influence of age group (five categories) and gender on fatigue in the patients’ sample. The effects of clinical setting, tumor stage, metastases, and ECOG performance state on fatigue were tested with three-way ANOVAs with the cofactors age group and gender. Cronbach’s alpha was chosen to indicate the reliability of the scales. The associations between the fatigue scales and other dimensions of QoL were tested with Pearson correlations. Confirmatory factor analyses (CFAs) were calculated with Mplus. We tested the one-dimensional and the originally designed five-dimensional model of the MFI-20. In addition, we tested the short form MFI-10, also in terms of a one-dimensional model and a three-dimensional model according to Baussard et al. [25]. Several fit indices were examined to evaluate the overall fit of each model: The *χ*^2^ goodness-of-fit statistic, the comparative fit index (CFI), the Tucker–Lewis index (TLI), the standardized root mean square residual (SRMR)**,** and the root mean square error of approximation (RMSEA) according to Hu and Bentler [34]. The statistical analyses, except the CFAs, were performed with SPSS version 24.

## Results

### Sample characteristics

Because the MFI-20 was administered in only three of the study centers, only 1824 of the 4020 patients filled in the MFI-20, at least in part. We restricted the analyses to those participants who had at least three valid scores for each of the five scales. This resulted in 1818 patients with valid MFI-20 scores (Table 1).

**Table 1:** Characteristics of the cancer patients’ sample

	Males		Females		Total	
	(*n* = 825)		(*n* = 993)		*(n =* 1818)	
	*n*	%	*n*	%	*n*	%
Age (years)						
M (SD)	61.1	(10.1)	56.1	(11.6)	58.4	(11.2)
Marital status^a^						
Single	72	8.9	140	14.4	212	11.9
Married	642	79.4	605	62.1	1247	69.9
Divorced	69	8.5	134	13.8	203	11.4
Widowed	26	3.2	95	9.8	121	6.8
Education^a^						
< 10 years, basic secondary school	235	28.7	258	26.1	493	27.3
10 years; middle-level sec. school	234	28.6	372	37.6	606	33.5
> 10 years; high school graduate	349	42.7	360	36.4	709	39.2
Tumor						
Breast	4	0.5	399	40.2	403	22.2
Digestive organs	195	23.6	150	15.1	345	19.0
Male genital organs	271	32.8	0.0	0.0	271	14.9
Female genital organs	0	0.0	174	17.5	174	9.6
Respiratory organs	101	12.2	57	5.7	158	8.7
Blood, blood-forming organs	65	7.9	78	7.9	143	7.9
Urinary tract	87	10.5	46	4.6	133	7.3
Lip, oral cavity, pharynx	28	3.4	17	1.7	45	2.5
Skin	16	1.9	18	1.8	34	1.9
Mesothelial and soft tissue	15	1.8	16	1.6	31	1.7
Eye, brain, CNS	8	1.0	10	1.0	18	1.0
Other	35	4.2	28	2.8	63	3.5
Tumor stage, UICC^a^						
1	77	14.8	197	27.6	274	22.2
2	117	22.4	158	22.2	275	22.3
3	115	22.0	118	16.5	233	18.9
4	213	40.8	240	33.7	453	36.7
Metastases^a^						
No	435	69.2	588	70.8	1023	70.1
Yes	194	30.8	242	29.2	436	29.9
Clinical setting						
Inpatient	365	44.2	330	33.2	695	38.2
Outpatient	160	19.4	270	27.2	430	23.7
Rehabilitation	300	36.4	393	39.6	693	38.1
ECOG performance status^a^						
0: asymptomatic	328	42.7	447	47.4	775	45.3
1: symptomatic but ambulatory	299	38.9	355	37.6	654	38.2
2: symptomatic, < 50% in bed during day	119	15.5	115	12.2	234	13.7
3: symptomatic, > 50% in bed, not bed-bound	18	2.3	25	2.6	43	2.5
4: bed-bound	4	0.5	2	0.2	6	0.4
Surgery^a^						
No	197	24.3	161	16.5	358	20.0
Yes	614	75.7	817	83.5	1431	80.0
Radiation^a^						
No	561	69.2	502	51.3	1063	59.4
Yes	250	30.8	476	48.7	726	39.9
Chemotherapy^a^						
No	454	56.0	379	38.8	833	46.6
Yes	357	44.0	599	61.2	956	53.4
Hormone therapy^a^						
No	760	92.1	800	81.8	1560	87.2
Yes	51	6.2	178	18.2	229	12.8

^a^Missing data not reported

### Comparison between patients and the general population

**Fig. 1:**
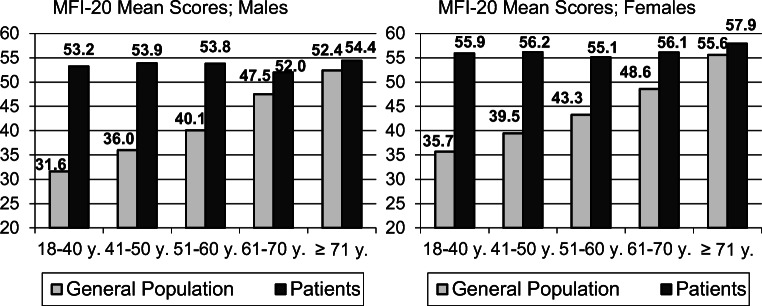
Mean scores of the MFI-20 total score for patients and general population, broken down by gender and age

Figure 1 presents the MFI-20 mean scores (sum scores) for the cancer patients and the general population. While in the general population there is a clear increase with increasing age, no such age trend was detected among the patients. In the age range 71 years and above, there are only marginal differences between the patients and the general population.

### Mean scores of the subscales and reliability coefficients

The mean scores of the five subscales, the MFI-10 total score, and the MFI-20 total score are given in Table 2. Female patients showed slightly higher fatigue mean scores (total *M* = 56.0) than males did (total *M* = 53.3). A comparable gender difference was found for each subscale of the MFI-20; the highest gender differences were found for the general fatigue and mental fatigue subscales. The ANOVA results reflecting the impact of gender and age group on the MFI-20 total score for the patients’ group were as follows: gender: *F* = 6.3, *p* = 0.012, age group: *F* = 0.599, *p* = 0.663, and interaction gender * age group: *F* = 0.418, *p* = 0.796.

**Table 2:** Mean scores of the MFI-20 scales including the sum scores of MFI-20 and MFI-10, comparison between cancer patients and general population, and reliability coefficients

	General fatigue	Physical fatigue	Reduced activity	Reduced motivation	Mental fatigue	MFI-20 sum	MFI-10 sum
Cancer patients							
Males							
*M*	11.6	12.0	11.7	8.8	9.1	53.3	25.3
(SD)	(4.4)	(4.6)	(4.5)	(3.6)	(4.2)	(17.9)	(9.5)
Females							
*M*	12.7	12.3	12.0	9.0	10.1	56.0	26.5
(SD)	(4.5)	(4.6)	(4.7)	(3.9)	(4.7)	(18.7)	(9.9)
Total							
*M*	12.2	12.2	11.9	8.9	9.6	54.8	26.0
(SD)	(4.5)	(4.6)	(4.6)	(3.8)	(4.5)	(18.4)	(9.7)
Effect size gender	0.25***	0.07 ns	0.07 ns	0.05 ns	0.23***	0.15***	0.12**
General population							
*M*	9.3	9.2	9.1	8.6	8.2	44.6	20.9
(SD)	(3.7)	(4.2)	(4.0)	(3.3)	(3.4)	(16.6)	(8.5)
Effect size patients—gen.pop.	0.71***	0.68***	0.65***	0.09***	0.35***	0.58***	0.55***
Alpha (patients)	0.84	0.86	0.87	0.71	0.86	0.94	0.89

**p < 0.01; ***p < 0.001; *ns*, not significant; *gen.pop.*, general population

The patients reported higher levels of fatigue than the general population did on all subscales. However, the differences were small in magnitude for reduced motivation (*d* = 0.09) and mental fatigue (*d* = 0.35), while the effect sizes were higher than 0.60 for the other three subscales (Table 2). The reliability coefficients of the subscales were between 0.71 and 0.87; the reliability of the MFI-20 total score was highest with alpha = 0.94 (Table 2).

### Clinical factors and fatigue

Table 3 presents the MFI-20 mean scores for the cancer types. The highest burden of fatigue was found among patients suffering from cancers of the blood and blood-forming organs, the skin, the category eye, brain, CNS, and mesothelial and soft tissue. Relatively low fatigue scores were reported by patients suffering from cancers of the male genital organs and breast cancer.

**Table 3:** The impact of tumor localization on fatigue

	*n*	General fatigue		Physical fatigue		Reduced activity		Reduced motivation		Mental fatigue		MFI-20 sum		MFI-10 sum	
		*M*	SD	*M*	SD	*M*	SD	*M*	SD	*M*	SD	*M*	SD	*M*	SD
Breast	403	12.1	4.5	11.1	4.5	11.0	4.7	8.6	3.7	10.0	4.6	52.7	18.5	24.9	9.5
Digestive organs	345	12.5	4.7	12.4	4.7	12.2	4.6	9.0	3.8	9.4	4.4	55.4	19.2	26.3	10.2
Male genital organs	271	10.4	4.2	10.8	4.3	10.5	4.1	8.1	3.2	8.7	4.0	48.6	16.7	23.1	9.0
Female genital organs	174	12.9	4.3	13.1	4.5	12.7	4.8	9.2	4.1	10.3	5.0	58.1	19.0	27.6	10.1
Respiratory organs	158	12.5	3.9	13.3	4.2	13.0	4.4	9.5	3.8	9.2	4.0	57.5	16.2	27.2	8.9
Blood, blood-f. organs	143	13.6	4.2	13.4	4.5	12.9	4.4	9.7	4.1	10.6	4.5	60.2	17.7	28.3	9.4
Urinary tract	133	12.3	4.3	12.3	4.6	12.0	4.4	9.0	3.5	9.0	4.1	54.7	17.1	26.6	9.3
Lip, oral cavity, pharynx	45	12.2	3.9	13.0	4.2	12.4	3.9	8.7	4.2	9.1	4.0	55.5	16.4	25.8	8.9
Skin	34	13.2	4.5	13.2	4.1	12.6	4.1	10.4	4.1	11.0	5.2	60.3	18.9	28.7	10.7
Mesothelial and soft tissue	31	13.5	4.1	14.3	4.6	14.0	4.9	9.5	3.1	9.8	4.5	61.0	15.9	29.3	8.9
Eye, brain, CNS	18	13.2	6.0	13.9	4.9	13.4	5.7	10.6	4.5	10.9	5.6	62.0	23.8	30.2	12.8
Other	63	11.7	4.4	12.3	5.0	11.2	4.9	8.7	4.1	9.7	4.3	53.7	18.9	25.7	10.2

The impact of the clinical setting (inpatient, outpatient, and rehabilitation), tumor stage, the presence of metastases, and the ECOG status on fatigue is given in Table 4. Fatigue increased with higher cancer stages, the presence of metastases, and higher scores in the ECOG level.

**Table 4:** The impact of the clinical setting and clinical variables on fatigue

	n	General fatigue		Physical fatigue		Reduced activity		Reduced motivation		Mental fatigue		MFI-20 sum		MFI-10 sum	
		*M*	SD	*M*	SD	*M*	SD	*M*	SD	*M*	SD	*M*	SD	*M*	SD
Setting		n.s.		***		***		n.s.		n.s.		*		*	
Inpatient	695	12.3	4.5	12.7	4.5	12.5	4.7	9.1	3.8	9.5	4.4	56.1	18.4	26.7	9.9
Outpatient	430	12.3	4.5	12.2	4.7	12.1	4.7	9.1	3.8	9.3	4.3	55.0	18.6	26.1	10.1
Rehabilitation	693	12.0	4.5	11.6	4.5	11.1	4.3	8.6	3.8	10.0	4.7	53.2	18.0	25.2	9.3
Tumor stage		***		***		***		**		n.s.		***		***	
1	274	11.7	4.4	11.1	4.4	10.7	4.4	8.6	3.9	9.6	4.4	51.8	18.4	24.5	9.4
2	275	11.8	4.6	11.4	4.5	11.1	4.3	8.6	3.6	9.5	4.5	52.4	18.0	24.7	9.3
3	233	11.7	4.4	11.8	4.6	11.6	4.4	8.7	3.6	9.4	4.3	53.2	17.6	25.3	9.2
4	453	13.1	4.4	13.3	4.5	13.1	4.7	9.6	3.9	9.9	4.5	58.9	18.3	28.2	10.0
Metastases		***		***		***		***		n.s.		***		***	
No	1023	11.9	4.5	11.7	4.6	11.4	4.4	8.7	3.7	9.6	4.4	53.3	18.0	25.2	9.4
Yes	436	13.0	4.5	13.2	4.6	13.0	4.8	9.6	3.9	10.0	4.5	58.9	18.6	28.1	10.1
ECOG performance		***		***		***		***		**		***		***	
0	775	11.4	4.5	11.0	4.5	10.8	4.3	8.3	3.5	9.3	4.4	50.8	17.9	24.0	9.4
1	654	12.5	4.4	12.5	4.4	12.0	4.6	9.0	3.8	9.7	4.5	55.8	18.0	26.6	9.6
2	234	13.9	4.0	14.4	4.2	14.4	4.1	10.0	3.9	10.1	4.4	62.8	16.0	29.8	8.9
3–4	49	14.4	4.4	15.5	3.5	15.3	4.1	11.2	3.8	10.8	4.1	67.2	16.1	32.4	9.1

*p < 0.05; ***p < 0.001

### Correlations between MFI-20 and other QoL scales

Among the 13 scales of the EORTC QLQ-C30, the three-item fatigue scale showed the highest correlation coefficients (between 0.49 and 0.77) with the MFI-20 scales (Table 5). Since the three items of the EORTC QLQ-C30 fatigue scale mainly indicate physical fatigue, the correlations with the MFI-20 scales general fatigue and physical fatigue are the highest. Of the five MFI-20 subscales, in most cases, general fatigue and physical fatigue showed the highest coefficients. Relatively weak associations were observed for the last two subscales, reduced motivation and mental fatigue, with the exception of the high correlation between cognitive functioning and mental fatigue (*r* = − 0.71).

**Table 5:** Correlations between the MFI-20 scales including the sum scores of MFI-20 and MFI-10 and the scales of the EORTC QLQ-C30

EORTC QLQ-C30 scales	General fatigue	Physical fatigue	Reduced activity	Reduced motivat.	Mental fatigue	MFI-20 sum	MFI-10 sum
Physical functioning	− .60	− .72	− .67	− .48	− .34	− .68	− .65
Role functioning	− .55	− .62	− .61	− .40	− .35	− .61	− .58
Emotional functioning	− .56	− .47	− .44	− .49	− .58	− .61	− .58
Cognitive functioning	− .48	− .39	− .40	− .42	**−** .71	− .58	− .55
Social functioning	− .51	− .54	− .51	− .41	− .41	− .57	− .53
Global health/QoL	− .60	− .68	− .62	− .47	− .37	− .66	− .62
Fatigue	*.77*	*.73*	*.69*	*.52*	*.49*	*.77*	*.75*
Nausea/vomiting	.30	.32	.29	.22	.18	.32	.32
Pain	.40	.47	.41	.31	.27	.45	.42
Dyspnea	.39	.40	.35	.27	.25	.40	.40
Insomnia	.38	.33	.30	.29	.34	.39	.36
Appetite loss	.38	.43	.42	.35	.24	.44	.42
Constipation	.16	.19	.17	.17	.12	.19	.19
Diarrhea	.21	.22	.19	.12	.12	.21	.22
Financial difficulties	.27	.26	.23	.19	.23	.28	.27
Sum score	− .71	− .73	− .68	− .55	− .55	− .77	− .74

Italic: correlations between the EORTC QLQ-C30 fatigue scale and the MFI-20 scales

When comparing the coefficients for the MFI-10 and the MFI-20 total scores, nearly all coefficients of the MFI-20 were slightly higher, in most cases with a difference of between 0.02 and 0.03.

### Factor analyses

Table 6 presents the CFA results. The one-dimensional model of the MFI-20 (model 1) shows the weakest fit coefficients. Considering the five dimensions (model 2) results in a remarkable improvement of the fit, though the coefficients CFI and TLI did not reach the thresholds for good model fit. The one-dimensional model of the MFI-10 (model 3) yielded better fit indices than the one-dimensional MFI-20. The last row in Table 6 takes into account the factorial structure of the MFI-10 (model 4); the resulting fit indices are marginally better than those of the MFI-20 when the five factors are taken into account (model 2).

**Table 6:** CFA fit indices

Model	Chi^2^ (df)	CFI	TLI	RMSEA	SRMR
M1: one-dimensional, MFI-20	4785.021 (170)	.778	.751	.127	.077
M2: five-dimensional, MFI-20	2014.696 (160)	.911	.894	.083	.045
M3: one-dimensional, MFI-10	971.583 (35)	.880	.846	.126	.059
M4: three-dimensional, MFI-10	558.945 (32)	.933	.905	.099	.045

## Discussion

The first aim of this study was to determine the burden of fatigue experienced by cancer patients. The cancer patients’ fatigue level was markedly higher than that of the general population. The effect size of the MFI-20 total score difference between the patients and the general population was *d* = 0.58, well above the criterion given by Norman et al. [35] who proposed adopting half a standard deviation (*d* = 0.50) as a criterion of clinical significance. Figure 1 clearly shows that there is a difference between the patients and the general population in terms of the effect their age had on their levels of fatigue. While we observed a clear link between increase of fatigue and increasing age in the general population, there was no statistically significant age effect in the patients’ sample. Clinicians should be aware that young cancer patients in particular suffer from fatigue compared with their healthy peers and that they need special support in the treatment of fatigue. This phenomenon, a nearly linear increase in the general population and small age effects in the patients, is not cancer-specific; it can also be found in other groups of patients [36]. However, in the Colombian general population study [14] the age trend was weaker than in the German normative study, and in the Swedish general population study [13], the age trend did not occur at all.

Concerning the five dimensions of the MFI-20, the most reliable and valid scales were general fatigue, physical fatigue, and reduced activity. The reliability of these scales was high with alpha coefficients above 0.80, and the differences between the patients and the general population (*d* > 0.60) were also the greatest on these scales. The lowest contribution was obtained from the scale reduced motivation which had an insufficient alpha coefficient and revealed only marginal differences between the groups (patients and general population).

Even though the MFI-20 with its five dimensions already covers a relatively broad spectrum of fatigue, qualitative studies show further characteristics of this issue. A recent meta-analysis of qualitative studies [37] identified six constructs in the sense of new interpretations of fatigue: embodied experience, (mis)recognition, small horizon, role changes, loss of self, and regaining one’s footing. Nevertheless, among the existing fatigue scales, the MFI-20 is one of the best at approaching these additional facets of fatigue.

The MFI-20 is well-suited to test the effects of acquiescence and response style because of the balanced proportions of positively and negatively oriented items [24]. The common variance of the equally oriented items is not reflected in the scale structure. Therefore, the differences in the item orientation result in a certain degree of unexplained variance which reduces the reliability of the scales. Therefore, it is interesting to test the MFI-10 which was constructed to omit such wording effects. The coefficients of the MFI-10 (reliability, effect sizes for the comparison between patients and general population, and correlations with the scales of the EORTC QLQ-C30) were slightly lower than those of the MFI-20, but they remained within an acceptable range. The shortening of the 20-item instrument to the 10-item version seems to be a good alternative for clinicians who are interested in using a shorter instrument.

As in other studies [10, 21, 38], the CFA fit indices of the MFI-20 were not satisfying. We did not create a new factorial structure for the MFI-20 as was done by other researchers since we believe that it is not useful to postulate new factors which make the results of different studies incomparable. The MFI-10 was however worth testing. The fit coefficients of the one-dimensional MFI-10 were better than those of the one-dimensional MFI-20, a result which can be interpreted as a consequence of having removed all of the items with an opposite direction [24]. Taking into account the subscale structure yielded better fit indices than those obtained with the one-dimensional models; the fit coefficients of the MFI-20 and the MFI-10 were of similar magnitude when the subscale structures were taken into account. Nevertheless, the reliability (Cronbach’s alpha) of the MFI-20 total score (0.94) was very good and higher than the coefficients of the MFI-10 total score (0.89) and the subscales. Moreover, the correlations of the MFI-20 total score with the scales of the EORTC QLQ-C30 were higher or at least as high as those of the MFI-20 subscales. Even when researchers and clinicians acknowledge that fatigue is a multidimensional construct, they are nevertheless often interested in having a summarizing score for fatigue. In such cases, both the MFI-20 total score and the MFI-10 total score are suitable measures.

There are no generally accepted cutoff scores for the MFI-20. In two studies, cutoff scores were used which were derived from a general population sample under the assumption that heightened fatigue means a score above the 75th percentile of the corresponding age and gender group [22, 29]. However, since this criterion is somewhat arbitrary, we preferred not to express the degree of fatigue in terms of persons above such a cutoff.

The impact of tumor type on fatigue is presented in Table 3. The lowest fatigue levels were found for cancers of the male genital organs, mostly prostate cancer. This result has also been found in several other studies [39]. Since the overall gender differences in fatigue were small in magnitude (*d* = 0.15), this effect can only partly be accounted for by the male gender factor.

The clinical setting (inpatient, outpatient, and rehabilitation) only had a small impact on the patients’ fatigue levels. As such, one can justifiably compare fatigue assessments obtained in these varying settings. As was to be expected, tumor stage, the presence of metastases, and the ECOG performance score were clearly associated with fatigue. With the exception of one subscale, mental fatigue, all of the MFI-20 subscales contributed to these differences. Thus, it is justifiable to evaluate the impact of these factors on the basis of a fatigue total score.

Some limitations of this study should be mentioned. It is possible that there was a certain selection bias. Patients suffering from severe fatigue might be underrepresented, which means that our mean scores might underestimate the actual burden of fatigue present in this patient group. The data of the general population comparison group is from 2003; however, since then, no normative study has been performed in Germany. We only tested the most important models with CFAs. We could have also tested other models proposed in the literature, e.g., three- or four-dimensional solutions. In addition, we could have calculated bifactorial models, including the total factor as well as the five single factors, which generally yield better fit indices. When we analyzed the impact of tumor site and other clinical variables on the fatigue levels, we only used bivariate statistics. Tumor type and other variables such as tumor stage may be correlated and confounded with age and gender.

In summary, fatigue is a severe problem among cancer patients. The MFI-20 proved to be an appropriate instrument for measuring fatigue, and the MFI-10 is a good alternative for clinicians interested in using a shorter questionnaire.
